# Microplastic fiber diet—Fiber-supplemented pellets for small fish

**DOI:** 10.1016/j.mex.2020.101204

**Published:** 2020-12-30

**Authors:** Anja Rebelein, Ulfert Focken

**Affiliations:** Thünen Institute for Fisheries Ecology, Bremerhaven, Germany

**Keywords:** Synthetic fibers, Polyester fiber, Exposure study, Microplastic toxicity, Fish feed, Dietary exposure

## Abstract

Ingestion of microplastic particles and fibers is frequently reported for aquatic organisms collected in the field. At the same time, only few studies investigate potential effects of ingestion of microplastic fibers due to handling issues in the laboratory. Exposure studies, which provide organisms with microplastic fibers via the diet, are a necessary step to analyze impact thresholds of vital and fitness parameters of aquatic organisms. Based on the limited number of studies providing fish with fiber-supplemented pellets, the following protocol presents a way to prepare a diet for fish that is supplemented with homogeneous distributed microplastic fibers for exposure studies. Produced pellets are suitable for small experimental fish, such as sticklebacks (2–5 cm), and can be manufactured up to amounts of several hundred grams and even few kilograms. The method can be adapted to different commercial fish feeds and microplastic fiber types due to manual preparation.

• Low-cost, manual preparation of microplastic fibers
• Preparation of a pelleted fish diet with uniformly distributed fibers
• Adaptable to different commercial fish feeds and microplastic fiber types.

## Graphical abstract

**Figure:**
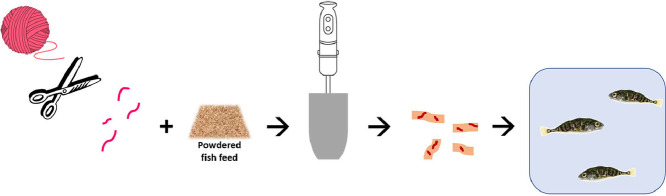

Specifications table

**Table:** 

Subject Area:	Environmental Science
More specific subject area:	Microplastic fiber exposure via dietary pellets
Method name:	Manual preparation of fish pellets containing microplastic fibers
Name and reference of original method:	n.a.
Resource availability:	n.a.

## Method details

### Background

Microplastic items (< 5 mm) are part of anthropogenic litter that are now ubiquitous in marine, freshwater and terrestrial ecosystems, and turned into an issue of global concern. Within the last decade increasing numbers of studies that investigate potential adverse effects of microplastic items on organisms were published. Provencher et al. [12] have recently called for more standardization in order to achieve repeatable methodologies, Barcelo [1] has stressed the importance of standardized analytical methods, and Barletta et al. [2] have presented a detailed sampling design to study microplastics in coastal and estuarine systems. However, in open systems such as estuaries, multiple stressors are present, and laboratory studies are necessary to identify their individual effects and the potential interaction between them. In aquatic environments the actual microplastic fiber contamination is suspected to be much higher than that of microplastic particles [3,^4^,7]. Microplastic fibers are even more difficult to handle than microplastic particles due to omni-present contamination issue, the entanglement and aggregation potential of fibers, and the lack of reference material – and were thus often neglected. The main challenges to overcome when handling microplastic fibers in the laboratory are exclusion of other airborne fibers, difficulties in weighing and counting the thin and irregular shaped fibers, and production of a homogeneous distribution of fibers in water and other matrices. Only a small number of studies were published so far that conducted exposure experiments with microplastic fibers provided via the diet [6,^8^,^9^,^11^,13]. While for gastropods and crustaceans inclusion of fibers in a biofilm or gelatinous matrix is possible, dietary pellets that contain microplastic fibers are more suitable for fish. Yet, manual insertion of fibers in fish pellets [8,11] is elaborate and only feasible for small number of pellets. Longterm-feeding of small fish (<3 g) already requires several gram feeds per fish (1 g equals 400–600 pellets), which cannot be manufactured by hand. Here, a protocol for the production of a fish diet (pellets), which contains an adjustable content of microplastic fibers in amounts that allow long-term feeding experiments with small fish, is presented. The described method was developed for a long-term exposure of juvenile stickleback (4–5 months old) with microplastic fibers via the diet. Pellets are prepared from a commercial fish feed that resembles natural food sources for sticklebacks. The pellets are supplemented with polyester fibers, which are a common fiber type in the textile industry [4] and in aquatic environments [7].

### Quality control

Glass and metal lab ware were used whenever possible. All equipment was thoroughly rinsed with ultra-pure water or filtered deionized water followed by a rinse with 96% ethanol to exclude microplastic contamination. Work was conducted under a laminar flow hood and every workspace was cleaned with ethanol before work. A cotton lab coat and green disposable gloves were worn at all times and it was refrained from wearing accessories. The ethanol was pre-filtered through a Whatman (Typ 1) cellulose filter to remove potential microplastic fiber impurities. Utensils and products were kept covered whenever possible and tools observed for contamination prior to operations.

## Microplastic fiber preparation

### Material

• Microplastic fiber wool (e.g. autofluorescent polyester)
• Fine scissors
• Glass petri dish
• Metal/ ceramic bowls
• Test sieves made from stainless steel (e.g. 25 µm and 300 µm)
• Glass tubes with aluminum screw cap
• Metal spatula and tweezers
• Aluminum foil
• 96% Ethanol (filtered, in a spray bottle)
• Ultrapure water
• Fluorescence microscope (e.g. Nikon ECLIPSE, Ts2R-FL)
• Software NIS-Elements AR (Nikon, 5.02.00)

### Procedure

**Fig. 1:**
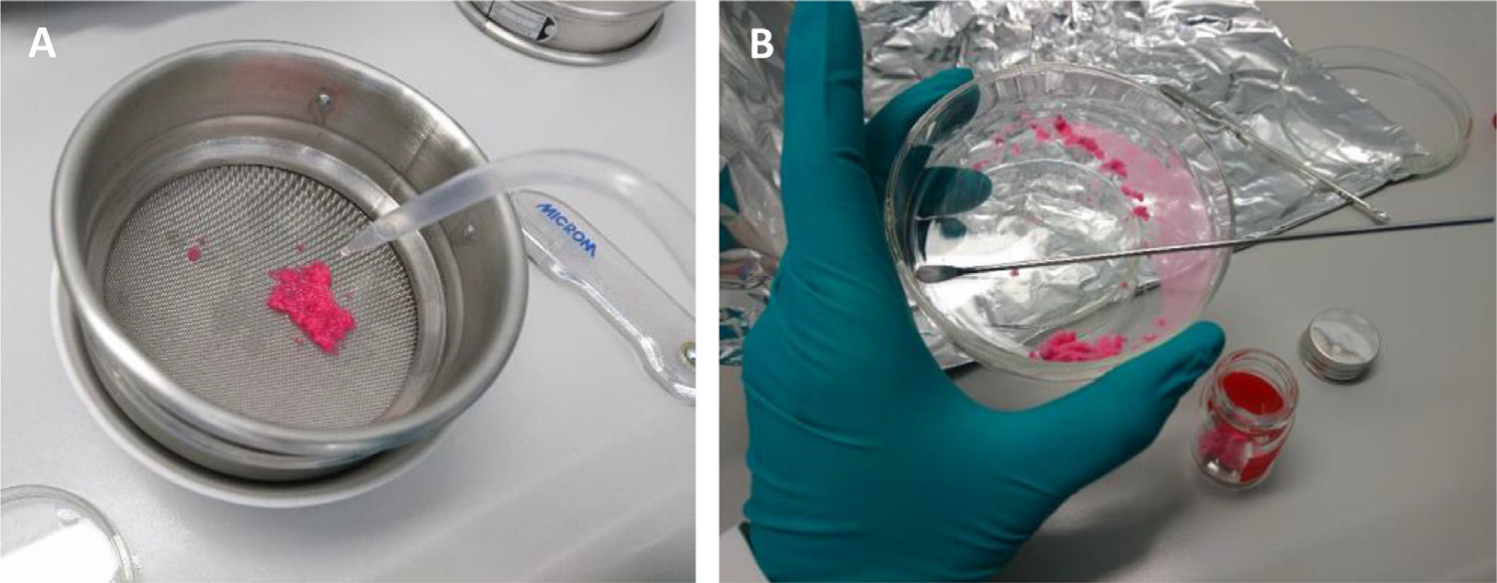
Preparation of microplastic fibers from commercial garment: sieving of cut fiber pieces through metal sieve (A) and collection of dried fibers into a glass tube (B). © Thünen-Institut/ Anja Rebelein.

Microplastic fibers were prepared in cleanroom facilities. Pink-red commercial polyester wool (Kuschelgarn, JES Collection, Germany) was used as raw material for the fibers. The garment was washed with water and ethanol to remove potential external dirt. Afterwards the threads were cut manually with scissors into small pieces. The process involves extensive cutting of folded wool sections for 45–60 min each. A high number of small-sized fiber pieces results from repeated chopping of the wool material. Cutting was done over a petri dish filled with some filtered ethanol to prevent that cut fiber pieces spread and distribute in the laminar flow hood due to electrostatic charging and instead stick to the petri dish after dropping. Moisten the garment with ethanol prior to cutting also helps to minimize fiber spreading. Extensive cutting of wool garment (mean 29.8 cm/ 292.2 mg per section) for 45–60 min releases about 159.5 mg small fiber pieces. Cut pieces were sieved twice through a 300 µm metal sieve and collected with a 25 µm metal sieve (Retsch, Germany) using pre-filtered ethanol (Fig. 1A) to narrow the fiber size spectrum of manual cut pieces. The respective filter mesh sizes were selected to create fibers that resemble the size range of polyester fibers released from textiles during washing (majority between 100 and 800 µm) [10]. However, this can be modified, and other mesh sizes facilitate the collection of different size fractions of fibers.

Collected polyester fibers were flushed with ethanol from the sieve into glass petri dishes. Petri dishes were left under the fume hood to evaporate the ethanol overnight. A loose cover with aluminum foil restricted potential airborne contamination. Dried fibers were collected with metal spatulas and stored in (aluminum) screw capped glass tubes (schuett-biotec, Germany) (Fig. 1B). Fibers were kept dry for storage to prevent aggregation as happens in aqueous suspensions.

## Diet preparation

### Material

• Cut microplastic fibers (e.g. polyester)
• Commercial fish feed, fine powdered (e.g. Essence (0.2–0.3 mm), Alltech Coppens)
• Commercial blender with metal whisks
• Metal press with disc insert for 1 mm diameter diet
• Metal mixing beaker
• Glass beaker
• Glass pipette
• Sieve (0.6 mm mesh)
• Aluminum foil
• Metal tweezers
• Chopping knife
• Deionized Water
• 96% Ethanol (filtered, in a spray bottle)
• Analytical balance (e.g. Sartorius)
• Fluorescence microscope (e.g. Nikon ECLIPSE, Ts2R-FL)
• Software NIS-Elements AR (Nikon, 5.02.00)

### Procedure

The fish diet was prepared by adding commercial fish feed to cut microplastic fiber pieces. Fine powdered fish feed (Essence, Alltech Coppens, Netherlands (S1)) with 0.2–0.3 mm grain size was used, as this feed is designed for recirculating systems and features an Artemia alternative that supplies the nutrient demand of sticklebacks.

Test diets were prepared with 0.2 mg and 2 mg fibers per g of fish feed (Table 1). Plastic fiber amounts (dry cut pieces) for about 50 g of feed were weighed into glass vials using an analytical balance (Sartorius, Germany) and suspended in pre-filtered ethanol. Microplastic fibers are easier to suspend and keep separate in ethanol than water, and aqueous suspension with surfactant should be avoided for fish diets. The fiber suspension was transferred to a metal mixing beaker (WMF, Germany) and the vial was rinsed twice with ethanol to transfer the total fiber amount. The ethanol suspension facilitates an equal distribution of fibers, which form a thin layer on the bottom of the mixing beaker. Fiber spreading with as little overlay as possible is necessary to ensure that fibers do not clump during mixing and distribute homogeneously within the diet. Depending on the size of the mixing container and the targeted final fiber concentration, only certain amounts of fibers can be processed at once. With the described setup (mixing beaker with 63.6 cm^2^ bottom surface), a maximum of about 150 mg fibers or 50–100 g of feed (depending on the fiber concentration) can be produced in one step.

**Table 1:** Ingredients for 50 g fish diet supplemented with 0.2 mg and 2 mg microplastic fibers per gram commercial feed (Essence).

Ingredients	Polyester Fibers	Essence feed	Deionized water
**0.2** **mg/g diet**	0.01 g	50 g	21.5 ml
**2** **mg/g diet**	0.1 g	50 g	21.5 ml

**Fig. 2:**
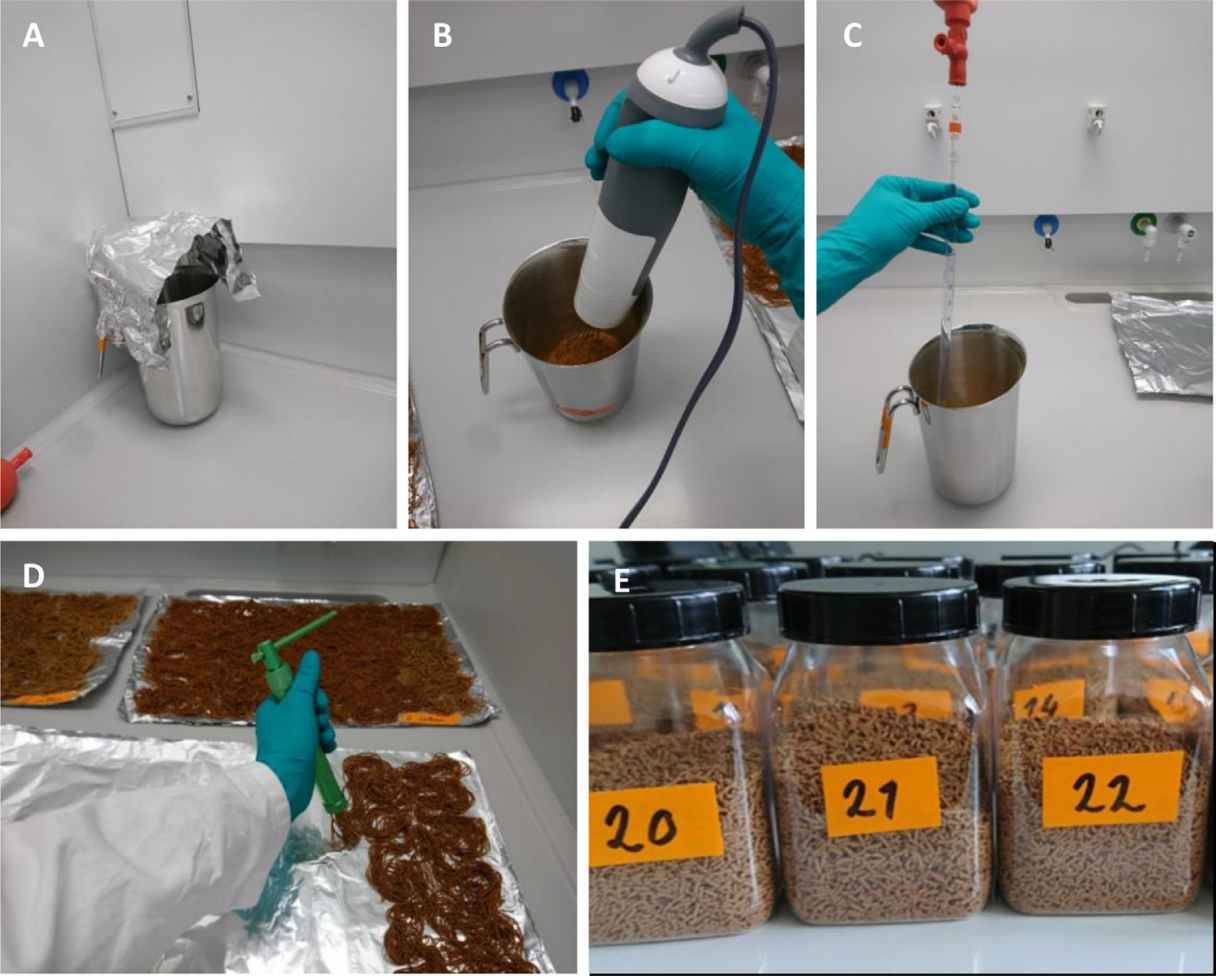
Preparation of the fish diet: evaporation of ethanol (A), mixing of dry components (B), addition of deionized water (C) and production of diet strings with a mechanical press (D). Dried feed pellets with and without fibers (E). © Thünen-Institut/ Anja Rebelein.

The fiber suspension was left to dry in the fume hood loosely covered with aluminum foil over night to evaporate the ethanol completely (Fig. 2A). Commercial fish feed (1 g per 0.2/ 2 mg fibers weighed) was added on top of the spread-out fibers and the dry components mixed thoroughly with a commercial blender (Fig. 2B). The well-mixed dry mass was supplemented with deionized water to form a homogeneous dough that could be pressed into feed strings (Fig. 2C, Table 1). A water content of 43% (v/w), which results in a just formable diet dough, was determined as optimum for the Essence feed and used setup. A higher water content would lead to a smoother dough, but results in more dense and harder pellets after drying, which are difficult to ingest for the fish. The feed strings were produced with a mechanical press (“Sugar paste extruder”, LIHAO, China) and had a diameter of 1 mm (Fig. 2D). The feed was left loosely covered with aluminum foil to dry in the fume hood overnight. Dried feed was crushed manually and with a chopping knife into pellets which can be easily ingested by the fish (1–5 mm in length). The pellet mix was sieved (0.6 mm mesh) to exclude powder and broken pieces smaller than 0.6 mm when feeding experimental sticklebacks.

## Characteristics of prepared fibers and diet

### Characterization of the fibers

Characterization of the polyester fibers was conducted using a Nikon fluorescent microscope (ECLIPSE, Ts2R-FL, Japan). The garment showed strong auto fluorescence in the red fluorescence filter (excitation 511–551 nm; emission 573–613 nm), while less auto fluorescence is visible with the green filter (excitation 490–510 nm; emission 520–550 nm) and no auto fluorescence could be detected with the DAPI filter (excitation 382–392 nm; emission 430–480 nm) (S2). Other fibers, such as fibers from paper towels used in the laboratory, did not fluorescent as intense in the red filter but did show strong fluorescence in the DAPI filter and were thus clearly distinguishable.

For analysis, a fiber suspension was prepared with dried fibers (0.05 mg/mL) in ultrapure water with 0.001% (v/v) Tween-80 surfactant solution (Merck, CAS-Nr. 9005–65–6) to ensure even dispersal of microplastic fibers without fiber aggregation. Low concentrations of the surfactant prevent the development of foam during homogenization of the solution when the glass vial is inverted multiple times, which would lead to uneven distribution of fibers.

**Fig. 3:**
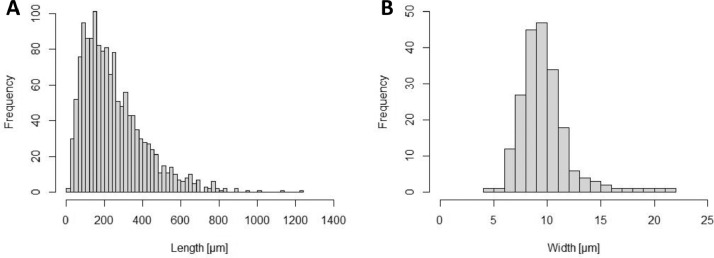
Polyester fiber length (*N* = 1446) (A) and width (*N* = 206) (B) distribution of manual cut pieces after sieving.

The suspension was pipetted onto microscope slides and analyzed using the software NIS-Elements AR (Nikon, 5.02.00). Mean polyester fiber length was 245.6 ± 163.5 µm (*N* = 1446) and fiber widths was 9.7 ± 2.3 µm (*N* = 206). Sieving narrows down the fiber size spectrum (length) to a certain extent (Fig. 3). Yet, the fiber diameter is well below 300 µm and some longer fibers slip through the meshes.

Pre-experimental trials revealed manual cutting to be the best comminution technique for the used polyester fibers as these fibers are soft and flexible. Mechanical cutting as e.g. described by Cole [5] might be used as well, but relies on the availability of specific lab equipment. Furthermore, the use of grinders and mechanical blades work better for more stiff plastic fiber materials than polyester, such as nylon. Depending on the fiber material used, mechanical cutting could facilitate a more homogeneous size distribution if that is desired for the experimental design.

### Verification of diet homogeneity and diet variations

**Fig. 4:**
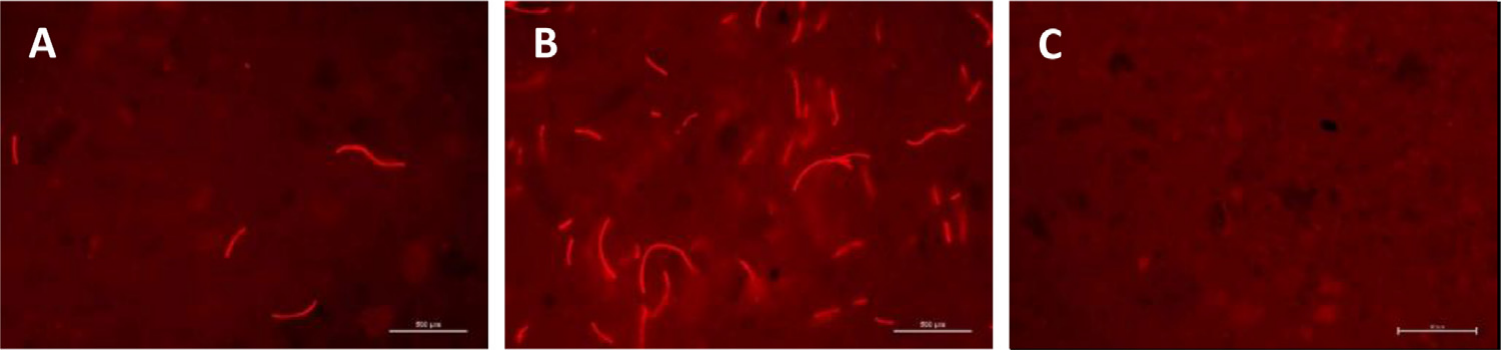
Homogenous spread of polyester fibers in produced pellets with 0.2 mg (A) and 2 mg (B) fibers per gram feed. Control pellets without fibers produced with pure Essence diet powder (C). © Thünen-Institut/ Anja Rebelein.

Homogeneity of the diets was verified by fluorescence microscopy of moistened and flattened pellets. Fibers are clearly visible within the diet (Fig. 4A and B) and thorough mixing ensures a homogeneous spread of the fibers. Control of repeated production lots demonstrated a homogeneous distribution of the fibers in all batches. Once inserted in the tanks, the diet sinks to the bottom of the tanks (5.3 cm/s mean sinking velocity) and sticklebacks feed on it in the water column and on the bottom. Submersed pellets soak some water but remain pelletized for more than an hour (S3), which is similar for feed with or without fibers.

Other commercial feeds can be used as basis as well if different nutrient requirements need to be supplied. Potential commercial feeds should be checked prior to the diet preparation to have low auto fluorescence in the red filter in order to easily identify and check the homogeneity of added fibers (Fig. 4C). Commercial feeds can be powdered or grain feed, but bigger-sized grains should be grinded prior to diet preparation to obtain a fine powder that ensures a homogeneous distribution of fibers. The amount of water necessary to form a smooth dough that is optimal for extrusion might vary depending on the nutritional composition and must be determined for each commercial feed separately. As outlined above, should the consistency of the prepared dough be just wet enough to be pressed with a mechanical press. Other types of microplastic fibers might be used as well provided that they show (at least low) fluorescent signals to confirm uniform distribution within the pellets.

## Appendix Supplementary materials

Image, application 1
