# Supplementary material for: Microplastic fiber diet—Fiber-supplemented pellets for small fish
Source: MethodsX. 2020 Dec 30;8:101204. doi: 10.1016/j.mex.2020.101204 (PMC8374235; doi:10.1016/j.mex.2020.101204)
Supplement: Supplementary file 1 [file mmc1.docx]

**Supplementary material *and/or* Additional information:**

S1. Composition and Energy content of the Essence Feed, as stated by Alltech Coppens.

| **Compound** | **Amount** |  | |
| --- | --- | --- | --- |
| Protein | 45 % |  |  |
| Fat | 11 % |  |  |
| Crude Fiber | 1.3 % |  |  |
| Ash | 7.2 % |  |  |
| Total Phosphor | 2.06 % |  |  |
| Vitamin A | 14 000 IE/kg |  |  |

| **Energy Content** | |
| --- | --- |
| Gross energy | 16.5 MJ/ kg |
| Digestible Energy | 14.8 MJ/ kg |


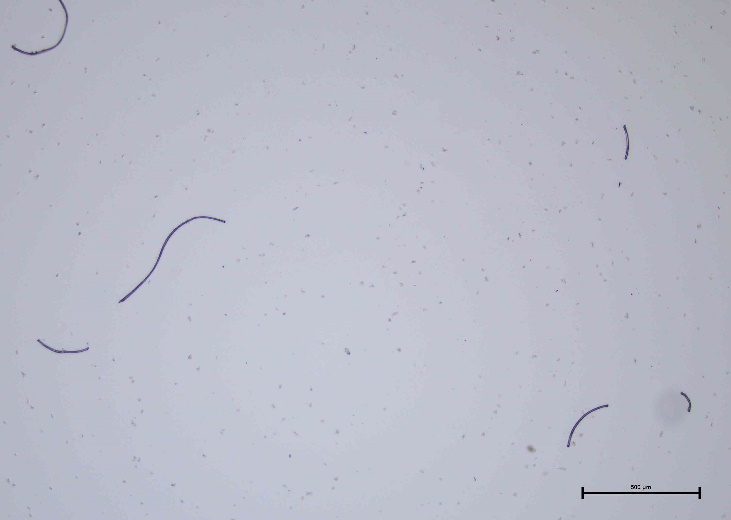
  
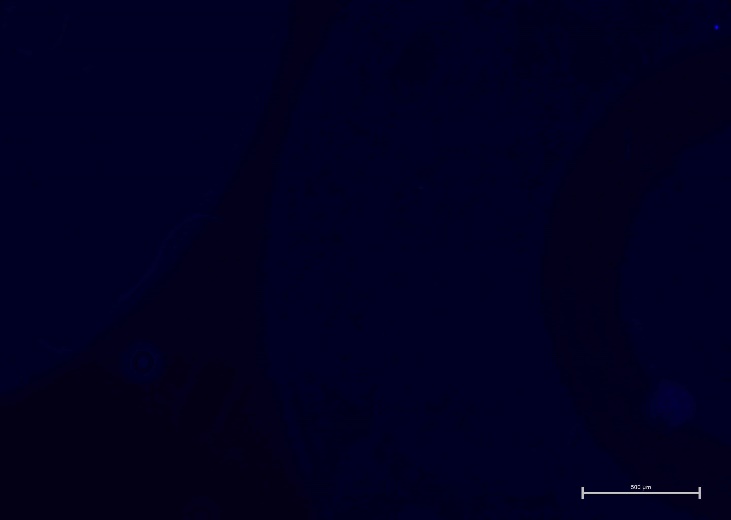

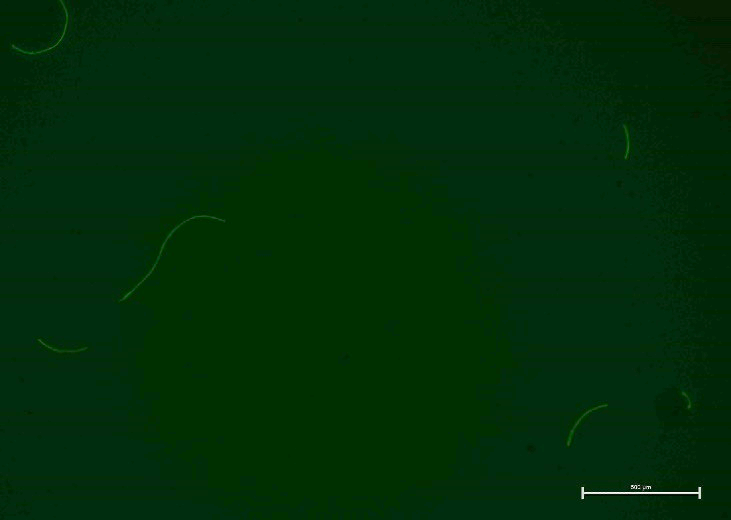
 
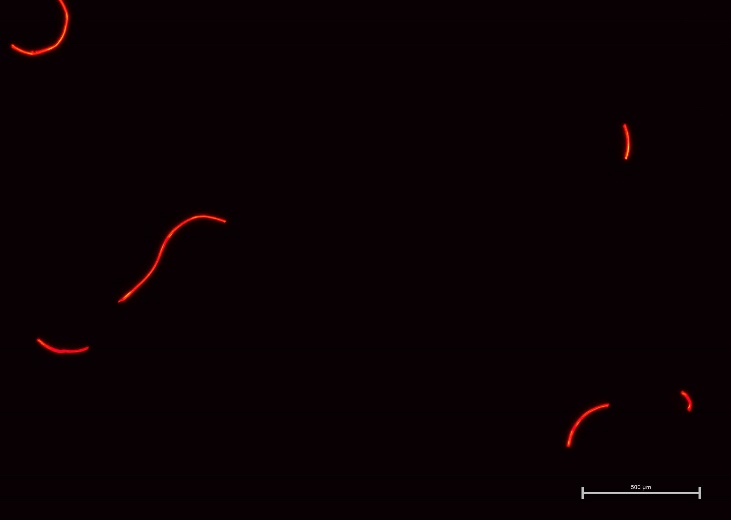


**B**

**D**

**C**

**A**

S2. Polyester fibers under a microscope, viewed with transmitting light (A), with a DAPI (B), green (C) and red (D) fluorescence filter captured with 500 ms illumination time (scale bar = 500µm). © Thünen-Institut/ Anja Rebelein


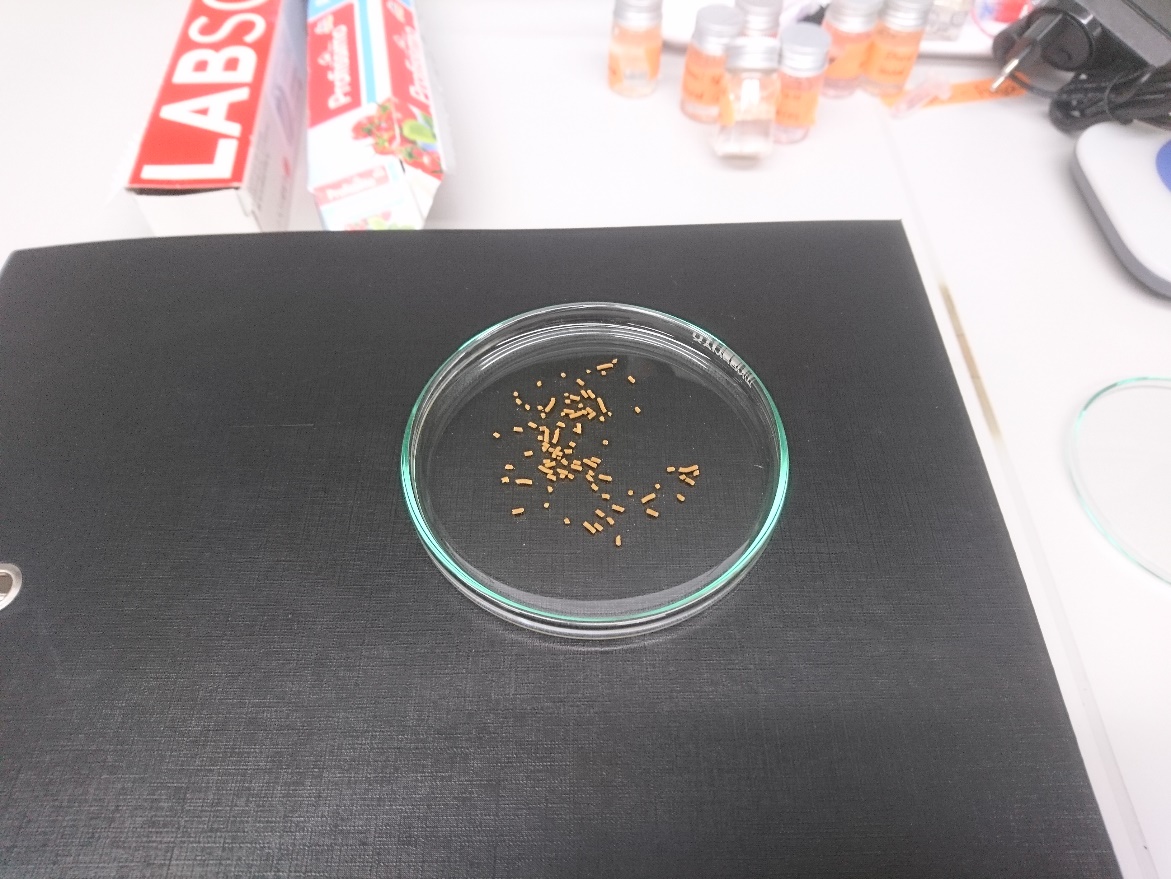

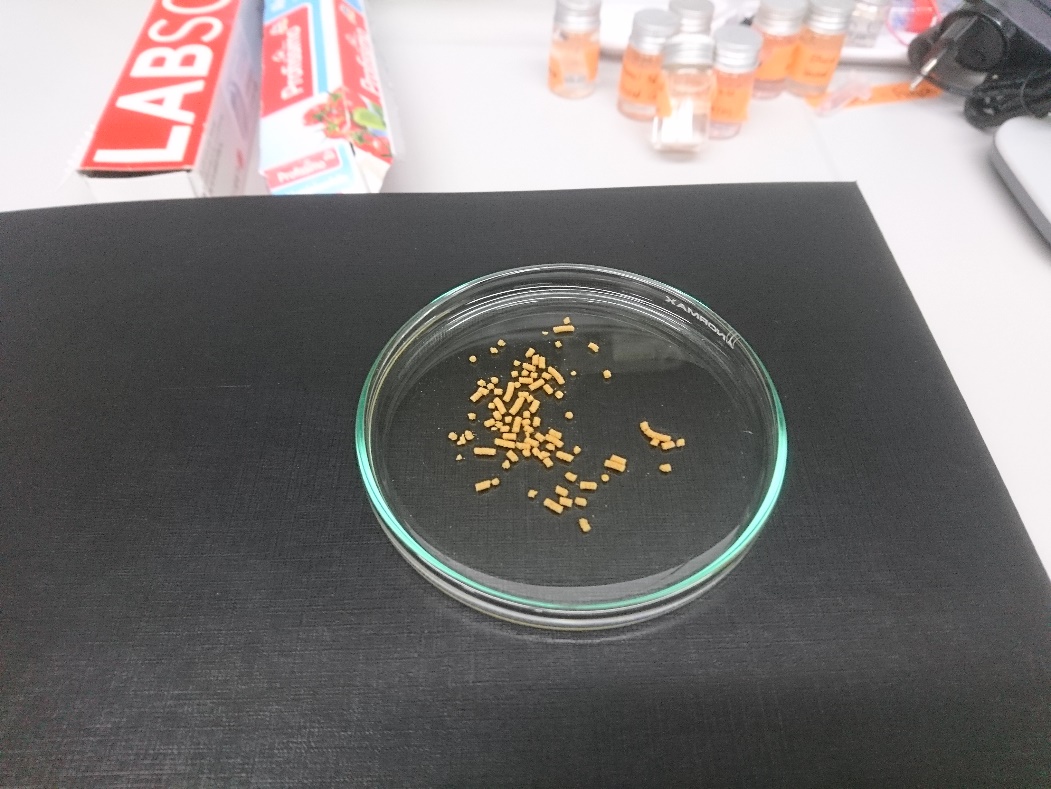

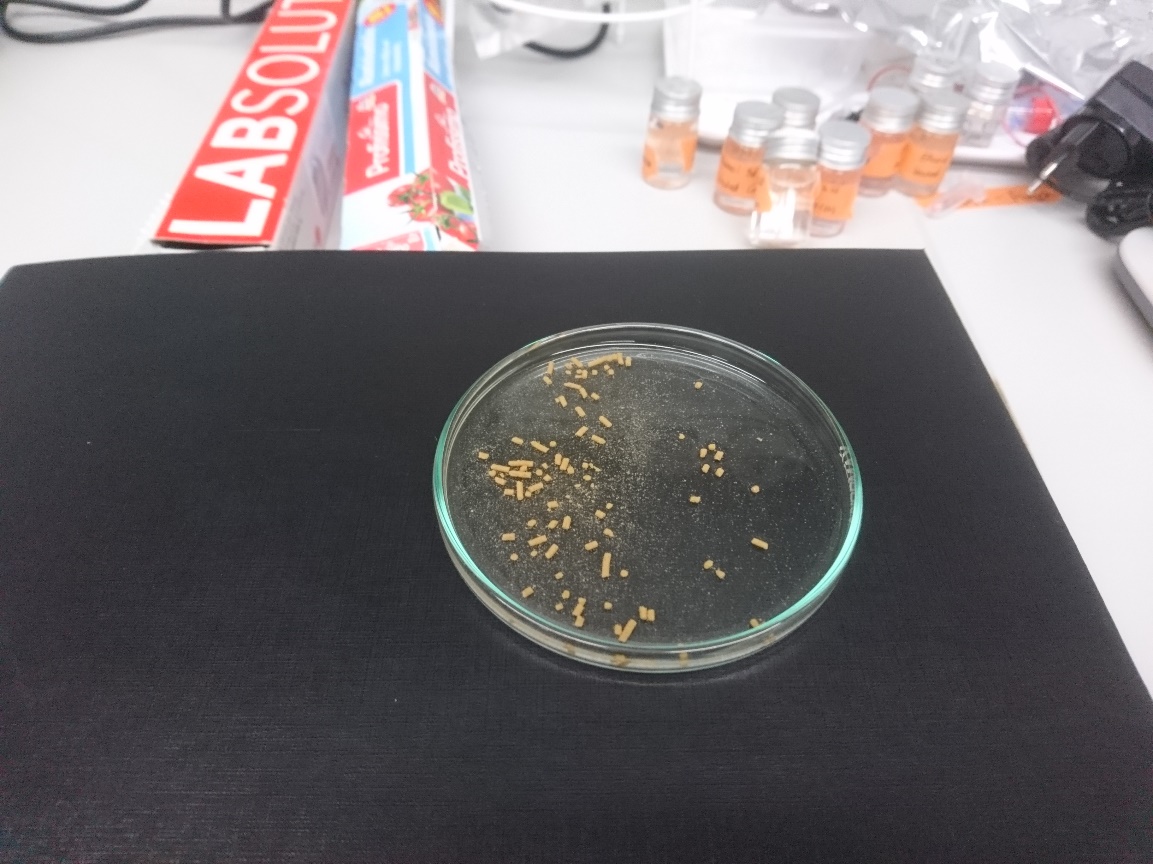


**C**

**B**

**A**

S3. Produced diet placed in water (A), after 60 minutes (B) and after 90 minutes and gentle shaking (C). Petri dishes are 9 cm in diameter. © Thünen-Institut/ Anja Rebelein
